# Ancestral Reconstruction and Investigations of Genomic Recombination on some Pentapetalae Chloroplasts

**DOI:** 10.1515/jib-2018-0057

**Published:** 2019-12-20

**Authors:** Christophe Guyeux, Michel Salomon, Bashar Al-Nuaimi, Bassam AlKindy, Jean-François Couchot

**Affiliations:** FEMTO-ST Institute, UMR 6174 CNRS, DISC Computer Science Department, Université de Bourgogne Franche-Comté, Besançon, France; Department of Computer Science, University of Diyala, Baqubah, Iraq; Department of Computer Science, University of Mustansiriyah, Baghdad, Iraq

**Keywords:** Ancestral genome reconstruction, Genomic recombination, Pentapetalae chloroplasts, Dynamic programming

## Abstract

In this article, we propose a semi-automated method to rebuild genome ancestors of chloroplasts by taking into account gene duplication. Two methods have been used in order to achieve this work: a naked eye investigation using homemade scripts, whose results are considered as a basis of knowledge, and a dynamic programming based approach similar to Needleman-Wunsch. The latter fundamentally uses the Gestalt pattern matching method of sequence matcher to evaluate the occurrences probability of each gene in the last common ancestor of two given genomes. The two approaches have been applied on chloroplastic genomes from *Apiales*, *Asterales*, and *Fabids* orders, the latter belonging to *Pentapetalae* group. We found that *Apiales* species do not undergo indels, while they occur in the *Asterales* and *Fabids* orders. A series of experiments was then carried out to extensively verify our findings by comparing the obtained ancestral reconstruction results with the latest released approach called MLGO (Maximum Likelihood for Gene-Order analysis).

## 1 Introduction

Chloroplasts are one of the main organelles in plant cells. They are considered to have originated from cyanobacteria through endosymbiosis, when an eukaryotic cell engulfed a photosynthesizing cyanobacterium, which remained and became a permanent resident in the cell.

Chloroplasts have the ability to convert water, light energy, and carbon dioxide (*CO*_2_) in chemical energy by using carbon-fixation cycle. This key role explains why photosynthetic organisms are at the basis of most trophic chains and are thus responsible for evolution and speciation. Moreover, as photosynthetic organisms release atmospheric oxygen when converting light energy into a chemical one, and simultaneously produce organic molecules from carbon dioxide, they are at the origin of the breathable air and represent a mid to long term carbon storage medium. Consequently, exploring the evolutionary history of chloroplasts is of great interest, and we propose to investigate it by the mean of ancestral genomes reconstruction. This reconstruction will be achieved in order to discover how the biomolecules (proteins and DNA) have evolved over time due to mutations or recombination. It will be useful to compute the mutation rate, and to determine whether evidences of their cyanobacteria origin can be presented in this way.

This article thus aims at exploring the possibility to reconstruct the Last Universal Common Ancestor (LUCA) of all available chloroplastic genomes, for a large variety of reasons encompassing the comparison with cyanobacterial genomes. The goal of this paper is not to provide a definitive answer to this ambitious question, but to investigate scientific and technical obstacles that may potentially appear when trying to reach such a difficult goal. In this proposal, the ancestral reconstruction has been achieved in two stages. Firstly, after having obtained a large collection of complete chloroplastic genomes, their coding sequences have been extracted and automatically annotated following the approach detailed in [1], [2]. Using the genes shared in common by these species, a well-supported phylogenetic tree has been obtained. However, the core genome of the whole species is too small to produce an accurate tree. A strategy was then applied which consisted in grouping subsets of sequences according to their similarity, inferring their phylogenies, and then merging the whole forest of trees, following the approach investigated in [3], [4], [5]. This first step being achieved, the second stage was to design algorithms that study the evolution of gene content and ordering among the supertree, and the latter must be validated with the naked eye on well chosen plant families. Our proposal in this article focuses on this second stage, and illustrates the kind of results that can be obtained on three small groups of *Pentapetalae* species. They have been selected due to the fact that, at the time of this study, only these groups of organisms had a sufficient number of complete genomes of good quality on NCBI. It will be completed in future work, by obtaining ancestral nucleotide sequence of each gene, and by filling intergenic regions using either state-of-the-art or novel algorithms.

Ancestral genome reconstruction has already been investigated several times in the literature [6], [7]. Over the past decade, many methods have been exploited to reconstruct phylogenies from gene-order data. The first algorithm of this kind was established by Fitch [8]. It assumed a binary alphabet and is based on the maximum parsimony (MP) approach. It finds the label to the internal nodes of a tree that reduces the number of changes or modifications along tree edges. Usually, state-of-the-art algorithms deal with the permutations of integers, each integer corresponding to the position of the associated gene in the lexicographic ordering of the pan-genome gene names. In other words, tools like Badger [9] do not support genomes of various lengths and with repeated/missing genes. Our problem applied to chloroplasts may appear as more difficult, as we relax the permutation hypothesis. However, in the classical Multiple Genome Rearrangement Problem [10], targeted genomes are either bacterial or nucleus ones, which have many more genes than a chloroplast. Furthermore, gene order and content do not evolve so much when considering related plant species. Such observations explain why state-of-the-art algorithms cannot be applied to our particular problem even if this latter should be solvable. Note that a new tool, called MLGO, can be applied for phylogenetic and ancestral genome reconstruction regarding gene-order data. MLGO relies on two methods: MLWD [11] for phylogenetic analysis and PMAG+ [12] for ancestral genome reconstruction. This tool uses the advantage of binary encoding on gene-order data, supports a relatively general model of genomic evolution (including not only rearrangements but also gene insertions, deletions, inversions, and duplications), and successfully accommodates itself into the framework of maximized likelihood.

## 2 Presentation of the problem

Let us consider a set of complete chloroplastic genomes for close plant species, like the ones presented in Table 1.

**Table 1:** Genomes information of all considered species.

**Organism name**	**Accession**	**NCBI Nucleotide database ids**	**Sequence length, pb**	**Number of genes**	**Order**	**Lineage**
*Ageratina adenophora*	NC_015621.1	334701780	150,698	183	Asterales	Eupatorieae
*Anthriscus cerefolium*	NC_015113.1	323149061	154,719	166	Apiales	Apiaceae
*Aralia undulata*	NC_022810.1	563940258	15,6333	169	Apiales	Araliaceae
*Artemisia frigida*	NC_020607.1	470227687	151,076	164	Asterales	Artemisiinae
*Brassaiopsis hainla*	NC_022811.1	558602891	156,459	168	Apiales	Araliaceae
*Castanea mollissima*	NC_014674.1	313183972	160,799	165	Fabids	Fagaceae
*Castanopsis echinocarpa*	NC_023801.1	595789916	160,647	165	Fabids	Fagaceae
*Chrysanthemum indicum*	NC_020320.1	452849029	150,972	167	Asterales	Artemisiinae
*Chrysanthemum morifolium*	NC_020092.1	441403271	151,033	165	Asterales	Artemisiinae
*Chrysobalanus icaco*	NC_024061.1	630716125	162,775	166	Fabids	Chrysobalanaceae
*Corynocarpus laevigata*	NC_014807.1	317046152	159,202	165	Fabids	Corynocarpaceae
*Cucumis melo*	NC_015983.1	346578170	156,017	163	Fabids	Benincaseae
*Cucumis sativus*	NC_007144.1	68164782	155,293	168	Fabids	Benincaseae
*Daucus carota*	NC_008325.1	114107112	155,911	166	Apiales	Apiaceae
*Eleutherococcus senticosus*	NC_016430.1	359422122	156,768	169	Apiales	Araliaceae
*Fragaria chiloensis*	NC_019601.1	428697178	155,603	137	Fabids	Fragariinae
*Fragaria vesca subsp. bracteata*	NC_018766.1	408830224	129,788	164	Fabids	Fragariinae
*Fragaria vesca subsp. vesca*	NC_015206.1	325126844	155,691	164	Fabids	Fragariinae
*Fragaria virginiana*	NC_019602.1	428697264	155,621	164	Fabids	Fragariinae
*Glycine canescens*	NC_021647.1	526176043	152,518	159	Fabids	Glycine
*Glycine cyrtoloba*	NC_021645.1	526175872	152,381	159	Fabids	Glycine
*Glycine dolichocarpa*	NC_021648.1	526176130	152,804	159	Fabids	Glycine
*Glycine falcata*	NC_021649.1	526176217	153,023	159	Fabids	Glycine
*Glycine max*	NC_007942.1	91214122	152,218	160	Fabids	Glycine
*Glycine soja*	NC_022868.1	558604116	152,217	160	Fabids	Glycine
*Glycine stenophita*	NC_021646.1	526175959	152,618	159	Fabids	Glycine
*Glycine syndetika*	NC_021650.1	526176311	152,783	159	Fabids	Glycine
*Glycine tomentella*	NC_021636.1	520850563	152,728	159	Fabids	Glycine
*Guizotia abyssinica*	NC_010601.1	183217719	151,762	161	Asterales	Millerieae
*Helianthus annuus*	NC_007977.1	94502469	151,104	161	Asterales	Heliantheae
*Hevea brasiliensis*	NC_015308.1	326909372	161,191	169	Fabids	Micrandreae
*Jacobaea vulgaris*	NC_015543.1	334702303	150,689	166	Asterales	Senecioninae
*Kalopanax septemlobus*	NC_022814.1	563940364	156,413	169	Apiales	Araliaceae
*Lactuca sativa*	NC_007578.1	81176229	152,765	160	Asterales	Lactucinae
*Licania alba*	NC_024064.1	630716317	162,467	166	Fabids	Chrysobalanaceae
*Licania heteromorpha*	NC_024062.1	630716224	162,833	166	Fabids	Chrysobalanaceae
*Licania sprucei*	NC_024065.1	630716416	162,228	167	Fabids	Chrysobalanaceae
*Manihot esculenta*	NC_010433.1	169794052	161,453	164	Fabids	Manihoteae
*Metapanax delavayi*	NC_022812.1	558602979	156,343	169	Apiales	Araliaceae
*Panax ginseng*	NC_006290.1	52220789	156,318	169	Apiales	Araliaceae
*Pentactina rupicola*	NC_016921.1	377829855	156,612	170	Fabids	Spiraeeae
*Phaseolus vulgaris*	NC_009259.1	139387430	150,285	165	Fabids	Phaseoleae
*Populus alba*	NC_008235.1	110227059	156,505	161	Fabids	Saliceae
*Populus trichocarpa*	NC_009143.1	134093177	157,033	162	Fabids	Saliceae
*Praxelis clematidea*	NC_023833.1	598324104	151,410	191	Asterales	Eupatorieae
*Prunus kansuensis*	NC_023956.1	628249143	157,736	165	Fabids	Amygdaleae
*Prunus persica*	NC_014697.1	313183801	157,790	165	Fabids	Amygdaleae
*Pyrus pyrifolia*	NC_015996.1	346683273	159,922	165	Fabids	Maleae
*Ricinus communis*	NC_016736.1	372450118	163,161	167	Fabids	Acalypheae
*Schefflera delavayi*	NC_022813.1	558603067	156,341	170	Apiales	Araliaceae
*Trachelium caeruleum*	NC_010442.1	170784721	162,321	160	Asterales	Campanulaceae
*Trigonobalanus doichangensis*	NC_023959.1	609253093	159,938	159	Fabids	Fagaceae
*Vigna radiata*	NC_013843.1	289066804	151,271	162	Fabids	Phaseoleae
*Vigna unguiculata*	NC_018051.1	393396080	152,415	162	Fabids	Phaseoleae

**Figure 1:**
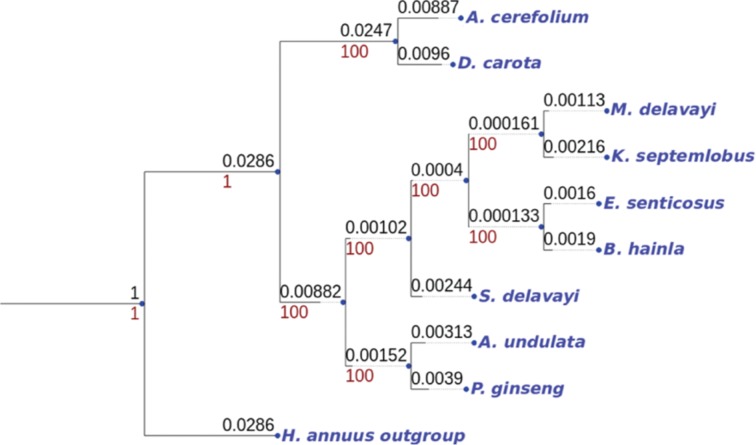
Most supported phylogenetic tree obtained from *Apiales* order. In this figure, the values at the top of the lines represent the branch lengths, while the values at the bottom correspond to the bootstrap supports (when it makes sense).

First, we assume that:

1. Each genome has been annotated with Dogma [13]. By doing so, the same gene prediction and naming process has been applied with the same quality of annotation. In particular, when a gene appears twice in the considered set of genomes, it receives twice the same name. At this level, each genome is described by an ordered list of gene names, with possible duplications. Other approaches are possible, see, *e.g.* [2], [14], [15].
2. The sequences inside the core genome (genes present everywhere in the considered set of species) have been multialigned, and a well supported phylogenetic tree has been obtained based on this alignment as shown in Figure 1 for *Apiales* order. This stage may necessitate the deletion of a few core genes that possibly blur the phylogenetic signal (for various reasons encompassing homoplasy, incomplete lineage sorting, horizontal gene transfers, *etc.*), for instance by using methods detailed in [1], [4], [5].

Our objective is then to reconstruct ancestral genomes at each node of the phylogenetic tree until the root node (last common ancestor). Such a reconstruction requires to first find the ordered list of genes of each ancestor, then the DNA sequence of each ancestral gene, and finally to fill in intergenic regions.

For all three steps of reconstruction, a set of authorized operations are provided, which are:

– insertion, deletion, duplication, or inversion of one or a block of genes, at gene lists level;
– operations commonly considered in the Needleman-Wunsch edit distance [16] (insertion, modification, or deletion of a nucleotide, together with opening and enlarging a gap), at DNA sequence levels.

The operations listed above allow a parsimonious approach to be used so that the number of leaf nodes is as small as possible. Note that the global optimum over the tree may be obtained with a few local solutions (one ancestor of two genomes) that are not optimal.

## 3 Ancestral analysis methods

Two methods have been applied on our set of data: an automatic Gestalt pattern based gene features matching process and a naked eye manual cross-validation. Let us begin by introducing the manual approach. This was completed first to determine which ancestor genomes our automatic algorithm should produce.

### 3.1 Method I: Naked eye investigation

**Figure 2:**
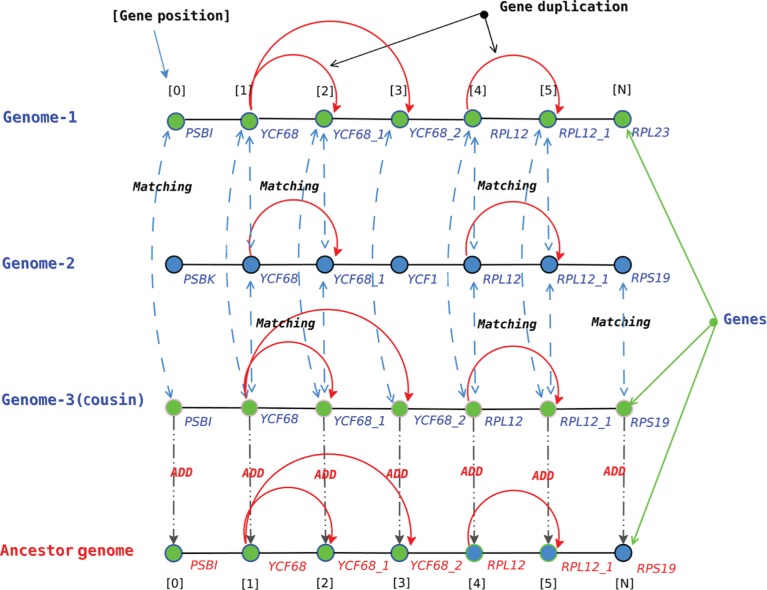
Simulation of ancestral reconstruction process between two genomes. ADD means that the considered sequence has been added to the list of genes in the ancestral genome.

As stated above, this method is not an algorithm that automatically builds the ancestors of the provided genomes, but it is a method applied manually, as follows. Some python codes were produced in order to graphically represent each triplet constituted by two sister species and their closest cousin as three parallel lines, as described in Figure 2 (the three lines at the top of the figure). On each line are located numerous equidistant vertices, one per coding sequence in the associated genome, and all the sequences having the same gene name according to Dogma are linked by a red edge.

The ancestral genome of each couple of sister species has then been manually deduced by a consensus approach. Each part shared in common in its genomes is put in the ancestor (see, for instance, the cases of YCF68 and YCF68_1: they are both in Genome-1 and Genome-2, so they were put on the ancestor). In case of a difference, both brother genomes are compared locally with their closest cousin. If the latter agrees with one of the two brothers, the agreement (sequence of genes) is put on the ancestor and this part of the lines is considered as resolved. For instance, PSBI is in Genome-1 but not in Genome-2. As it is in Genome-3, the parcimonious hypothesis is that it was in the ancestor of the 3 species, and that Genome-2 lost it.

If the closest cousin cannot help us to resolve the situation, because the cousin presents locally a third pattern different from the two brothers, then one or more new close cousins are considered recursively. It is the solution that reduces the largest number of rearrangement operations that is finally chosen, leading to the local ancestor gene list (again a parsimonious approach).

**Figure 3:**
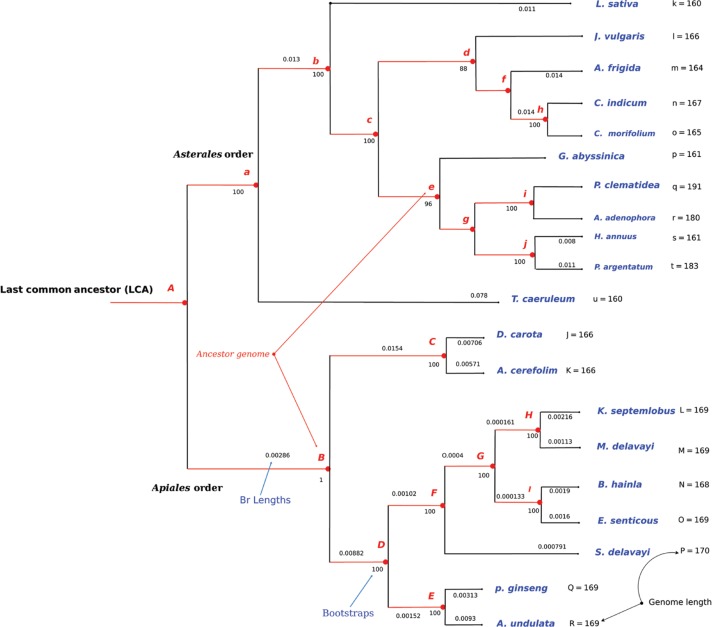
Names of the ancestors in the *Apiales* and *Asterales* phylogenetic tree, provided with supports and branch lengths: the numbers shown at each branch are bootstrap scores computed by RAxML [4], while branch lengths are indicated (cf. Br Lengths). A letter has also been associated to each internal node (the ancestors are in red), while the number of genes per genome is indicated under the label ”Genome length”.

By doing so and verifying our results three times, a trustworthy ancestral list of genes at each internal node was obtained. Our next objective was to recover these ancestors automatically.

### 3.2 Method II: Ancestor Prediction based on Gene Contents

**Figure 4:**
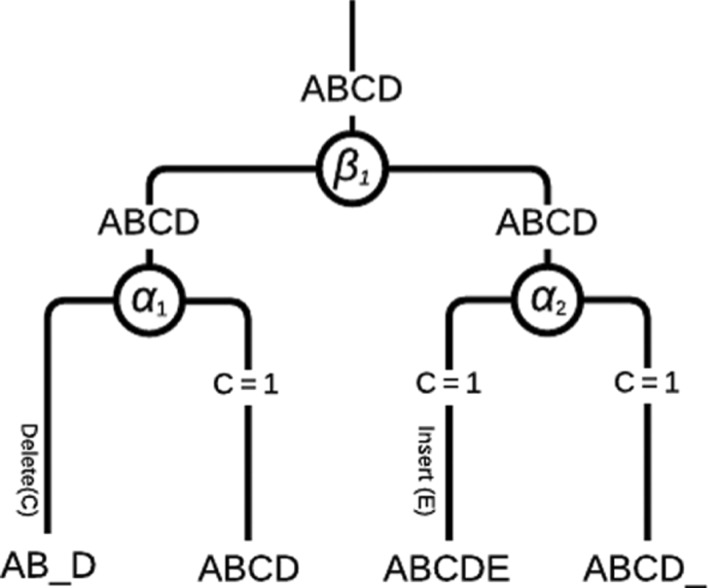
Simulation of gene investigation step between two genomes

**Figure 5:**
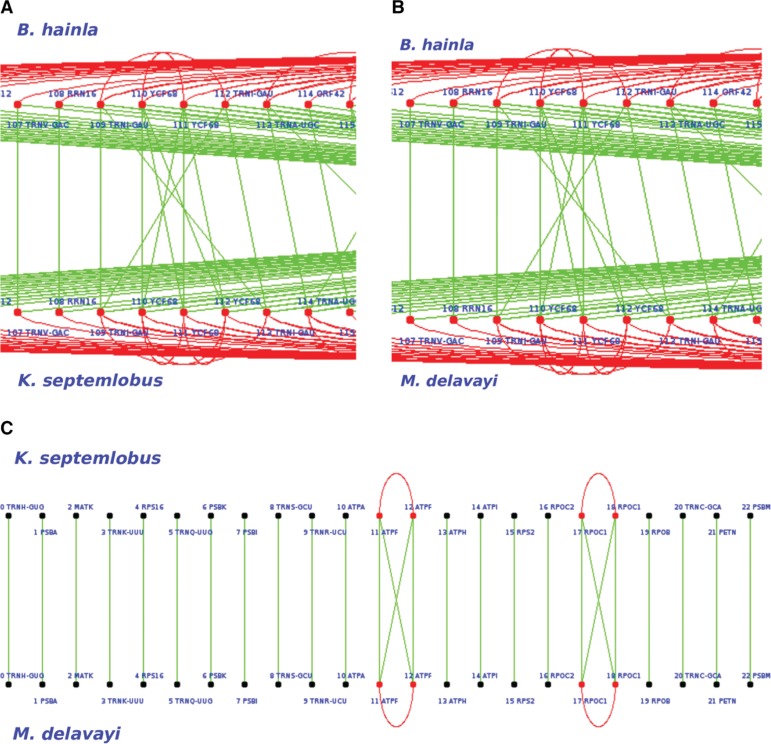
Graphical presentation of genes alignment between two genomes, useful in the naked eye investigation. Red curves indicate duplicated genes within a genome, while green lines indicate homology (same gene) between two distinct genomes. (A) At this particular location of the genome, a gene loss can be seen in *B. hainla* (or one gene gain in *K. septemlobus*), namely the YCF69. (B) As the same situation occurs between *B. hainla* and *M. delavayi*, we can conclude to a gene loss (as gaining twice the same gene at the same location is less likely). (C) At this location, the gene order is not modified between the genomes of the two considered sister species, namely *K. septemlobus* and *M. delavayi*.

This method encompasses the following general steps:

– **Step 1: Preliminary stage.** In this step, all internal nodes from leaf nodes to the root one are named following the alphabetical order. Each letter in an internal node represents an ancestor genome. This latter can be the ancestor of two leaves, of an internal ancestor and a leaf node, or of two internal ancestors. The result of this step can be seen in Figure 3. In this tree, a bottom-up procedure was applied to predict the ancestor genome at each internal node.
– **Step 2: Genome Selection.** Figure 3 presents the most supported topology for two *Campanulids* subgroups. The algorithm starts by automatically selecting the two closest sister species according to the Needleman-Wunsch distance applied to lists of genes. The other species are then ordered according to their distance in the tree (number of nodes between it and one of the two sister species, and Needleman-Wunsch distance to solve ex-aequo cases), defining what can be called an ordered list of cousins.
– **Step 3: Genes Investigation.** In the easiest situation, all gene couples completely match between the two sister species (which thus have the same length). In this case, the frequency of occurrences depends on the considered family, the ancestor is easily deduced as being the same as its children. If there is at least one problematic situation between the selected genomes (that is, if there is at least one deleted, duplicated, or inserted gene in one genome), as for example between *E. senticosus* and *B. hainla*, then a deeper investigation is initiated using one or more cousin genome(s). For instance, in this example, *M. selavayi* and *K. septemlobus* will be considered first as cousin genomes to take the final decision in the treatment of such problematic situations.
  In this case, all genes are iterated in the considered two brother genomes *U*_1_ and *U*_2_, if gene *g_i_* in *U*_1_ matches properly in name, position, and orientation with $g^{\prime}_{i}$ in *U*_2_, then it is added in the ancestor genome *γ* at position *i*. Otherwise, consider the gene $g^{\prime\prime}_{i}$ at the same location in the first cousin genome: if *g_i_* or $g^{\prime}_{i}$ is equal to $g^{\prime\prime}_{i}$ then add the most frequent gene to the ancestor genome *γ* in position *i*, else this gene is considered as an insertion.
Figure 4 offers a simulation example of the considered procedure. In this figure, suppose that A, B, C, D, and E in the leaves are genes, and the objective is to predict the ancestor *α*_1_. Note that genes A, B, and D match in positions. Concerning the problematic C gene between these two genomes, a cousin will be needed to determine whether it is present in the *α*_1_ ancestor genome or not. One or both genomes in *α*_2_ subtree are considered to be cousin(s) to treat the problem of gene C. The two cousin genomes have one copy of gene C in their gene lists. According to our voting system, gene C will be in *α*_1_ ancestor and a delete operation is recorded (that is, AB_D). An insert state is also marked in *α*_2_ subtree, where gene E did not appear in either cousin genomes of *α*_1_ tree, nor in its brother. Such a deletion is illustrated in Figure 5a and b.
  The simple conflict resolution presented above has been refined by considering the Gestalt pattern matching method [17] based on dynamic programming like Needleman-Wunsch, as it is implemented in the SequenceMatcher method of the difflib Python library.
– **Step 4: Ancestor update.** After applying the previous step to all the genes of the sister species, their ancestor is then reconstructed. The subtree of the two brother genomes is then replaced by the list of genes of their ancestor.
– **Step 5: Loop-back.** Repeat from Step 2 until the final root ancestor is constructed.

So all matching genes are directly assigned to the ancestor. For non-matching genes, the process consists of the selection of a third genome, among the cousins according to the provided tree. The selected cousin is the closest one to the two considered genomes, according to the chosen distance. It is then compared to the two sister species for each non-matching gene, if the cousin agrees with one sister, then the considered gene is added to the ancestor.

## 4 Discussion

The whole process of ancestral gene order reconstruction was performed on three data sets, namely: *Apiales*, *Asterales*, and *Fabids*. Their phylogenetic relationship, obtained using RAxML [18] on multi sequence alignment of their core genes, is reproduced in Figure 3.

### 4.1 The *Apiales* order

Let us first consider the *Apiales* order for which both the phylogenetic relationship and the gene lists of leaves are known at the beginning of the study. We then apply the manual and the automatic approaches to infer ancestral states at each internal node of the tree. The results were convergent and lead to the following conclusions regarding the evolution of gene content among the tree. We focused first on the evolution of duplications. Table 2 (supplementary material) contains all duplicated genes among the *Apiales* order. For each duplication, the number of copies is specified as well. Let us now enter into details regarding the leaves of the phylogenetic tree shown in Figure 3.

**Figure 6:**
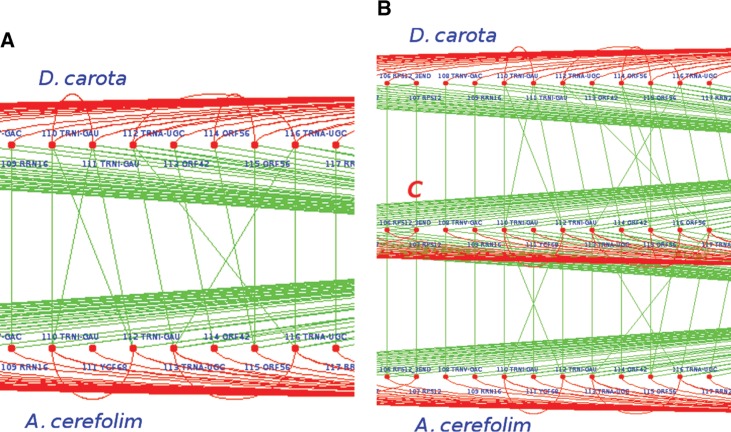
Green lines indicate homologuous genes while red vertices are for paraloguous ones. C line in figure (B) is the reconstructed ancestor of these two chloroplasts. (A) Example of gene correspondences in brother genomes D. carota and A. cerefolium. For instance *YCF*68 is found in position 111 in *A. cerefolium* while it is missing from *D. carota*. Additionally, *ORF*56 is in 2 copies, positions 114 and 115, in *D. carota*, while this gene is only represented once at position 115 in its sister. (B) Comparison between two brothers. The result is the ancestor genome *(C)*. We can reasonably deduce two copies of *ORF*56 have been deleted from genome *A. cerefolium*. The ancestor, for this part, contains also the gene *YCF*68, which has been deleted from the genome *D. carota*.

– Sister species *E. senticosus* and *B. hainla* have been considered first, with *K. septemlobus* playing the role of the cousin. After manual and automatic comparisons, we found that the gene *YCF*1 is present twice in *E. senticosus*, while it is in three copies in *B. hainla*. As the cousin has only two sequences of *YCF*1, we suggest that the latter is present twice in the ancestor, one gene has been inserted in *B. hainla*. Similarly, *YCF*68 is in 4 copies in *B. hainla* and in 6 copies in the sister species. As the cousin presents 6 copies as well, it can be deduced that the common ancestor of *E. senticosus* and *B. hainla* contains 6 copies of this gene. In other words, two copies of *YCF*68 have been removed in *B. hainla*. All the other genes are similar in both names and locations, and thus the ancestral genome *(I)* can be deduced.
– The brother genomes *A. undulata* and *P. ginseng* have exactly the same ordered list of genes, which is thus assigned to their last common ancestor *(E)*.
– Similarly, all couples of sister species *A. undulata* and *P. ginseng*, *M. delavayi* and *K.septemlobus*, *S.delavayi* and *M. delavayi*, and finally *K. septemlobus* and *E. sentucosus* match perfectly when considering each couple of brother genomes. In other words, they have not deviated from their respective last common ancestors, which presents the same sequence as their children species. Selected genomes are aligned graphically as shown in Figure 5. We then identify, by using naked eyes investigation and human thinking, the most parsimonious scenario applied on a deduced ancestor, which can lead to these two children using the lowest number of edit operations (such as inserted and deleted genes). Figure 5c shows this matching process applied on *M. Delavayi* and *K. septemlobus*, which have a core genome of 169 genes (only the 23 first genes are depicted). Note that, in this example, *M. Delavayi* (*L*) and *K. septemlobus* (*M*) match completely, so the ancestor *H* is very easy to obtain (*H* = *L* ∩ *M* has 169 genes).
– Let us finally compare *A. cerefolium* and *D. carota*. The *YCF*68 gene exists in 2 copies in *A. cerefolium* while it is missing in *D. carota*, as shown in Figure 6a. The cousin, for its part, also contains the gene *YCF*68 (in 6 copies), and so our algorithm concludes the presence of this gene (in 2 copies) in the ancestor of *A. cerefolium* and *D. carota*. Additionally, *D. carota* contains 4 copies of *ORF*56, while this gene is only represented twice in its sister. As the cousin genome has 4 representatives of *ORF*56, we can reasonably deduce that this is the case too in the ancestor of these two sister species: two copies of the gene *ORF*56 have been deleted from the genome *A. cerefolium*. Such decisions are depicted in Figure 6b, which shows a specific region of the ancestor genome *(C)*. This region has been generated by our algorithm, which has been applied on *A. cerefolium* and *D. carota*, and it has been manually validated.

**Figure 7:**
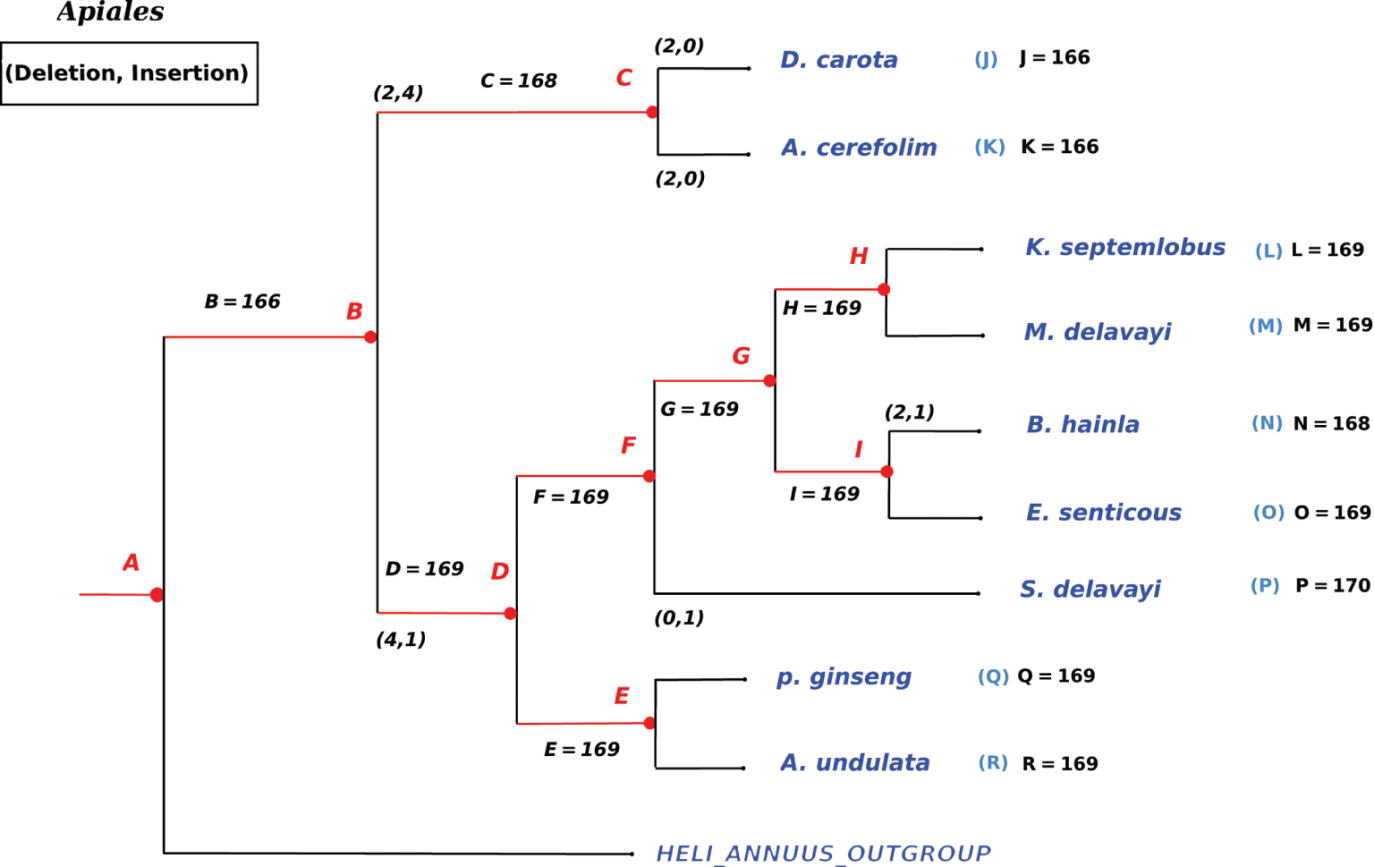
Insertion and deletion events found during ancestor reconstruction on *Apiales* order. Letters in red refer to ancestor genomes (their lengths are provided too).

The process detailed above continues with the obtained ancestors and is repeated until reaching the root of the tree: the Last Universal Common Ancestor (LUCA) of *Apiales*. By operating this reconstruction stage, we found that chloroplasts of this order have not faced so much deletion or insertion in their genomes. Indeed, in most cases, the disparity comes from the variation in numbers of gene copies. The obtained results are summarized in Figure 7.

### 4.2 The *Asterales* order

**Figure 8:**
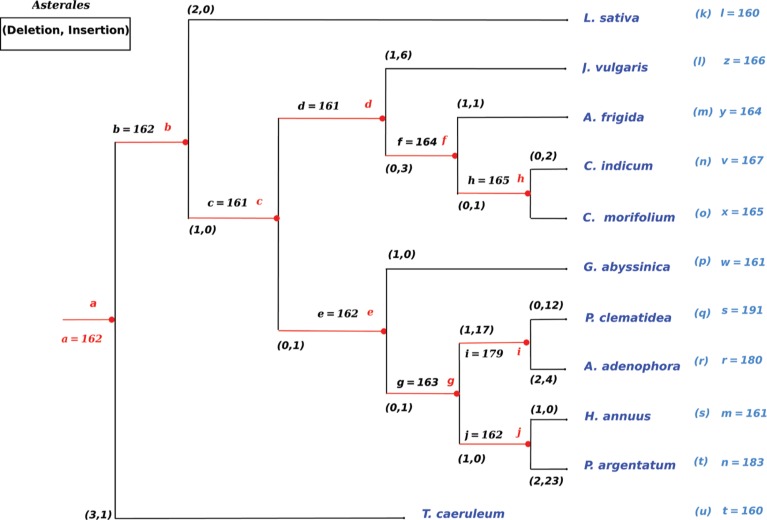
Summary of the complete ancestral genomes reconstruction of the *Asterales* order. Insertion and deletion events are provided, with names and length of each internal node.

The *Asterales* order, which is close to the *Apiales* one, was then examined. Table 3 in supplementary material contains what has been deduced from our experiments on this order. As can be seen, *Asterales* genomes have undergone many more changes compared to the *Apiales* ones. This difference between both orders lead to a larger variation in the lengths of *Asterales* genomes. For the sake of illustration, let us consider for instance the chloroplast of *H. annuus*. It only contains 161 coding sequences while its sister species, namely *P. argentatum*, has 183 genes. The matching process previously described has led in this case to an ancestor of size 162. More precisely, 23 genes have been inserted and two other ones have been deleted in *P. argentatum*, while only one gene has been removed in *H. annuus*, as described in Figure 8.

In this second order and in most cases, genes are comparable in both names and locations, having the same positions if we do not consider duplications. Indeed, almost all differences in this set of chloroplastic genomes come from a variation in the number of copies. Let us now investigate a third order, to compare it with *Apiales* (only a few variations of genomes) and *Asterales* (large variety in duplications).

### 4.3 The *Fabids* order

It was easy to deal with *Apiales* order, while *Asterales* improved the complexity of the ancestral reconstruction, due to duplications. However, in both cases the proposed algorithm was able to recover results that have been inferred manually (naked eye investigation). Let us now consider a larger and more complicated order, namely the *Fabids*, to evaluate the performances of our proposal when facing a complex collection of genomes.

Indeed, the main problem with this new order is that it contains large scale inversions in some branches, while it was not the case with both previously studied orders. In this case, a single inversion detection algorithm has been able to signal helpful information regarding such regions, like the beginning and the end (insertion or deletion) of reversals. However, the most difficult case where insertions or deletions are inside the inversion zone is difficult to handle.

**Figure 9:**
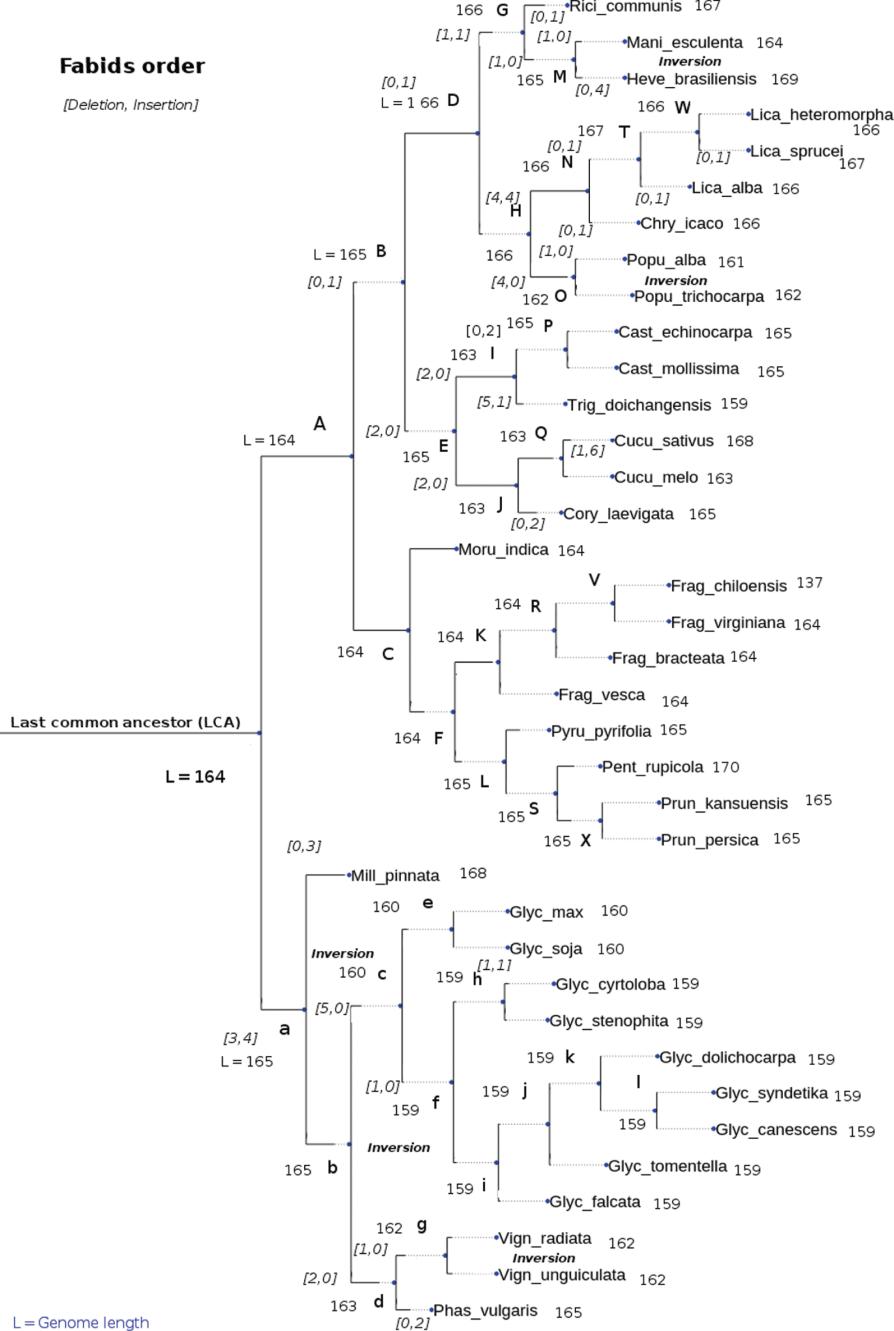
A phylogenetic tree in the reconstruction of the *Fabids* ancestor and the unambiguous reconstruction accuracies of our algorithms on this tree. Alphabetic characters represent the ancestors. *L* stands for the length of each node (number of genes), *[D, I]* describes the number of deletions and insertion, while inversions are also indicated.

In this situation, our proposition consisted in selecting one of the two brothers to operate as a reference. The best cousin was then searched within the same clade, and the status of each gene in this region (matching, or need insertion or deletion) was then compared. Most of the considered reversal regions match at the genes names level, but with reverse positions. Figure 9 presents our finding on *Fabids* order, with information about the length of each node. Various rearrangement information were also provided such as the number of insertion, deletion, and inversion.

**Figure 10:**
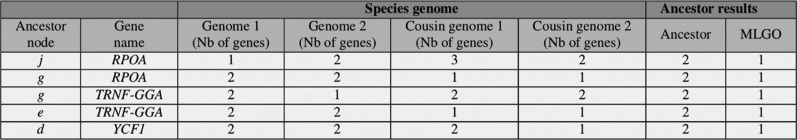
The variation in comparison results of ancestral genomes nodes on *Asterales* order with MLGO.

### 4.4 Comparison with MLGO

For the sake of comparison, we have examined the ancestral genome contents of both *Apiales* and *Asterales* species with MLGO tool, which stands for Maximum Likelihood for Gene Order Analysis1
http://www.geneorder.org.. This latter is, to the best of our knowledge, the first web tool for phylogeny and ancestral genomes reconstruction compatible with genome rearrangements [12].

On the one hand, the ancestors in the *Apiales* order, provided either by our approach or with MLGO, are very similar in terms of gene contents. However our method outperforms MLGO when investigating the specific location of genes and their number of duplications. On the other hand, the results are very different for some nodes in the *Asterales* case, as summarized in Figure 10. For instance, when gene *YCF*1 in internal node *d* has 2 copies in both its two children and their closest cousin has 2 occurrences of this gene too. In this case, our algorithm proposes to set the number of *YCF*1 in *d* to 2, while MLGO produced only one copy.

**Figure 11:**
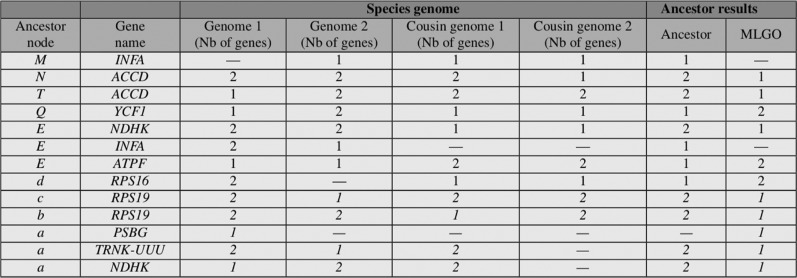
The variation in ancestral genomes nodes which were achieved by comparing our method results with *MLGO* tool on *Fabids* order.

Similar consequences can be outlined in the most difficult case, namely the *Fabids* order, as highligthed by Figure 11, each time, our algorithm outperforms the MLGO results by producing what is the most likely ancestral state (numbers of genes and their positions) in each situation. For instance, considering the ancestral node *M*, it was found that gene *INFA* is missing in the first child while it is present in the second one. It was also found in both its closest cousins, with one copy at each time. The most reasonable scenario is to consider that the ancestral node under consideration also has a single copy of *INFA*. This result is produced by our algorithm, while MLGO considers that *M* must not have *INFA* in its genome. Other divergent results can be reported, as in the ordinary case of node *E*: gene *ATPF* is present once in each of the two childrens. So our algorithm considers that it is present once in *E*, while with MLGO, this node must contain two copies of *ATPF*. Other nodes are problematic in the MLGO case, for example, {*a, b, c, d*} as can be seen in Figure 11. Each time, our algorithm produces results in agreement with the one that have been deduced manually, while in some cases MLGO has yielded surprising results. Indeed, these results were produced following a random extraction among the differences in the order of genes in the current species: on a large random extraction of our large set of reconstructed ancestral genes, leading to 18 different evolutionary scenarios according to our method and that of MLGO, we systematically discovered the right ancestral situation, while MLGO systematically misled itself.

**Figure 12:**
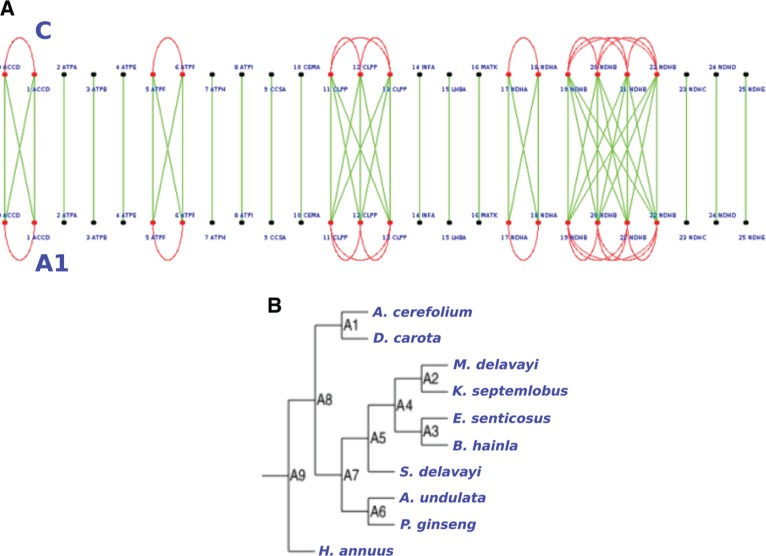
Green lines indicate homologuous genes while red vertices are for paraloguous ones. C line in figure (B) is the reconstructed ancestor of these two chloroplasts. (A) Example of comparison with MLGO on *Apiales* order. We show similarity in gene contents between our results, ancestor node *(C)*, and ancestor node *(A1)* from MLGO. (B) *Apiales* order tree produced by MLGO.

## 5 Conclusion and future works

Given a set of close annotated chloroplastic genomes, we have extracted the largest subset of core genes that lead to the most supported phylogenetic tree. On such trees, we have proposed a first ancestral reconstruction of gene content and order. The algorithm is based on the SequenceMatcher method of the Python difflib library and the results obtained were verified with the naked eye on well-defined families. Ways to merge the forest of phylogenetic trees in a supertree have been considered as well, and the way gene content evolves through a tree of core genomes has finally been presented.

This proposal belongs to an ongoing project regarding the design of the ancestral reconstruction of chloroplastic genomes. Our objective in this article was to show the feasibility of the approach on simple and specific events: duplication, insertion or deletion of a gene. More complex recombinations or larger magnitudes such as inversion, large-scale duplications, or events related to repeated sequences, certainly require further developments, and are beyond the scope of this article. But we intend to continue both the theoretical investigations and their applications. The next steps of such research work are to reconstruct the ancestral DNA sequences, to extend the algorithms to larger genomes (of bacteria, for instance), to apply them to larger sets of species (e.g. the whole available complete genomes of chloroplasts), and to extract various knowledge from these ancestors regarding the evolution of genome sequences.
