# Supplementary material for: Ancestral Reconstruction and Investigations of Genomic Recombination on some Pentapetalae Chloroplasts
Source: J Integr Bioinform. 2019 Dec 20;16(4):20180057. doi: 10.1515/jib-2018-0057 (PMC7074144; doi:10.1515/jib-2018-0057)
Supplement: Supplementary file 1 [file jib-16-20180057-s001.zip › j_jib-2018-0057_suppl/Supplementary.pdf]

Supplementary material

Table 2: Gene duplication for each genome in *Apiales* order.

	Genome name								
Gene name	*B. hainla*	*E. senticosus*	*A. undulata*	*P. ginseng*	*M. delavayi*	*A. Cerefolium*	*D. carota*	*S. delavayi*	*K. septemlobus*
ACCD	0	0	0	0	0	1	1	0	0
RPS12	1	1	1	1	1	1	1	1	1
NDHA	1	1	1	1	1	1	1	1	1
NDHK	1	1	1	1	1	0	0	1	1
RP12	3	3	3	3	3	3	3	3	3
RPS7	1	1	1	1	1	1	1	1	1
RPOC1	1	1	1	1	1	1	1	1	1
YCF2	3	3	3	3	3	3	3	3	3
YCF3	2	2	2	2	2	2	2	2	2
RPL23	1	1	1	1	1	1	1	1	1
YCF1	2	1	1	1	1	1	1	2	1
CLPP	2	2	2	2	2	2	2	2	2
ATPF	1	1	1	1	1	1	1	1	1
ORF56	3	3	3	3	3	1	3	3	3
RRN23	1	1	1	1	1	1	1	1	1
YCF68	3	5	5	5	5	1	0	5	5
RRN5	1	1	1	1	1	1	1	1	1
RRN4.5	1	1	1	1	1	1	1	1	1
YCF15	1	1	1	1	1	3	3	1	1
RRN16	1	1	1	1	1	1	1	1	1
ORF42	1	1	1	1	1	0	1	1	1
RPS19	0	0	0	0	0	1	1	0	0
TRNV-GAC	1	1	1	1	1	1	1	1	1
TRNL-UAA	1	1	1	1	1	1	1	1	1
TRNL-CAA	1	1	1	1	1	1	1	1	1
TRNV-UAC	1	1	1	1	1	1	1	1	1
TRNR-ACG	1	1	1	1	1	1	1	1	1
TRNN-GUU	1	1	1	1	1	1	1	1	1
TRNA-UGC	3	3	3	3	3	3	3	3	3
TRNI-GAU	3	3	3	3	3	3	3	3	3
TRNI-CAU	1	1	1	1	1	1	1	1	1
RPS12.3END	1	1	1	1	1	1	1	1	1

Table 3: Gene duplication for each genome in *Asterales* order.

	Genome name									
Gene name	*G. abyssinica*	*J. vulgaris*	*L. sativa*	*A. frigida*	*C. indicum*	*C. morifolium*	*P. clematidea*	*A. adenophora*	*H. annuus*	*P. argentatum*
ACCD	0	0	0	0	0	0	1	0	0	0
NDHB	3	3	3	3	3	3	3	3	3	3
RPS12	1	1	1	1	1	1	2	2	2	1
NDHA	0	0	1	1	0	0	0	0	0	0
NDHK	1	1	1	1	1	1	1	1	1	0
NDHF	0	0	0	0	0	0	0	0	0	1
RPL2	3	3	3	1	1	1	1	1	3	5
RPS7	1	1	1	1	2	2	2	2	1	3
YCF2	1	1	1	1	1	1	6	1	1	5
YCF3	2	2	2	2	2	2	2	2	2	2
RPL23	1	1	1	1	1	1	3	3	1	1
YCF1	0	0	0	0	1	1	3	2	0	3
CLPP	2	2	2	2	2	2	2	2	2	2
ATPF	1	1	1	1	1	1	2	1	1	1
ORF56	3	3	3	3	3	3	3	3	3	3
RRN23	1	1	1	1	1	1	1	1	1	1
YCF68	1	5	1	1	3	3	4	3	1	1
RRN5	1	0	1	1	1	1	1	1	1	0
RRN4.5	1	1	1	1	1	1	1	1	1	0
YCF15	1	3	1	1	1	1	1	1	1	0
RRN16	1	1	1	1	1	1	1	1	1	0
ORF42	1	1	1	1	1	1	0	1	1	0
RPS19	1	0	0	0	0	0	2	2	1	1
RPL14	0	0	0	0	0	0	1	1	0	0
RPS3	0	0	0	0	0	0	0	0	0	1
ORF188	0	0	0	0	0	0	0	0	0	0
CCSA	0	0	0	0	0	0	0	0	0	0
RPOB	0	0	0	0	0	0	0	0	0	2
RPOA	0	0	0	0	0	0	0	0	0	1
PSBD	0	0	0	0	0	0	0	0	0	2
TRNA-UGC	3	3	3	4	4	4	3	3	3	1
TRNI-GAU	3	3	3	3	3	3	3	3	3	3
TRNI-CAU	0	1	0	0	0	0	0	0	1	1
TRNI-ACG	1	0	1	1	1	1	1	1	0	0
TRNR-ACG	1	1	1	1	1	1	1	1	1	1
TRNN-GUU	1	1	1	1	1	1	1	1	1	1
TRNV-GAC	1	1	1	1	1	1	1	1	1	0
TRNL-UAA	1	1	1	1	1	1	1	1	1	0
TRNL-CAA	1	1	1	1	1	1	3	3	1	0
TRNV-UAC	1	0	1	1	1	1	1	1	1	0
TRNF-GAA	1	0	0	0	0	0	1	1	0	0
TRNT-GGU	0	0	0	1	1	1	0	0	0	0
TRAN-GCA	0	0	0	0	0	0	1	1	0	0
TRNS-GCU	0	0	0	0	0	0	1	2	1	1
TRNR-UCU	0	0	0	0	0	0	0	0	0	1
RPS12.3END	1	1	1	1	1	1	1	1	1	1
